# EMBRAVE: EMBedded Remote Attestation and Verification framEwork

**DOI:** 10.3390/s25175514

**Published:** 2025-09-04

**Authors:** Enrico Bravi, Alessio Claudio, Antonio Lioy, Andrea Vesco

**Affiliations:** 1 Dipartimento di Automatica e Informatica, Politecnico di Torino, 10129 Torino, Italy; enrico.bravi@polito.it; 2 Cybersecurity Research Group, LINKS Foundation, 10138 Torino, Italy; alessio.claudio@linksfoundation.com (A.C.); andrea.vesco@linksfoundation.com (A.V.)

**Keywords:** Remote Attestation, embedded system, IoT, cyber–physical system, TPM, Linux IMA, cybersecurity

## Abstract

The Internet of Things (IoT) is a growing area of interest with an increasing number of applications, including cyber–physical systems (CPS). Emerging threats in the IoT context make software integrity verification a key solution for checking that IoT platforms have not been tampered with so that they behave as expected. Trusted Computing techniques, in particular Remote Attestation (RA), can address this critical need. RA techniques allow a trusted third party (Verifier) to verify the software integrity of a remote platform (Attester). RA techniques rely on the presence of a secure element on the device that acts as a Root of Trust (RoT). Several specifications have been proposed to build RoTs, such as the Trusted Platform Module (TPM), the Device Identifier Composition Engine (DICE), and the Measurement and Attestation RootS (MARS). IoT contexts are often characterized by a highly dynamic scenario where platforms are constantly joining and leaving networks. This condition can be challenging for RA techniques as they need to be aware of the nodes that make up the network. This paper presents the EMBedded Remote Attestation and Verification framEwork (EMBRAVE). It is a TPM-based RA framework designed to provide a dynamic and scalable solution for RA in IoT networks. To support dynamic networks, we designed and developed Join and Leave Protocols, permitting attestation of devices that are not directly under the control of the network owner. This paper discusses the design and open-source implementation of EMBRAVE and presents experimental results demonstrating its effectiveness.

## 1. Introduction

The Internet of Things (IoT) [1] ecosystem is growing rapidly across several application fields [2,3,4]. IoT platforms can perform critical tasks [5,6,7], and compromising their integrity can lead to severe consequences. In some cases, such as Cyber–Physical Systems (CPS), the integrity of the devices is critical to ensuring the safety of the environment and people.

Remote Attestation (RA) [8] techniques can be adopted to verify the integrity of IoT platforms. RA is the process that allows a trusted third party (Verifier) to collect integrity evidence of a remote platform (Attester). Hardware-based RA techniques rely on a secure element on the platform that acts as a Root of Trust (RoT). The Trusted Platform Module (TPM) [9], proposed by the Trusted Computing Group (TCG) [10], is the most widely used secure element. Other specifications have been proposed for building RoTs on resource-constrained IoT platforms, such as the Device Identifier Composition Engine (DICE) [11] and the Measurement and Attestation RootS (MARS) [12]. In addition, several systems have been proposed that exploit the security properties and features of RoTs to measure the events that occur on an IoT platform. One of these is the Integrity Measurement Architecture (IMA) [13,14]. It is a Linux submodule responsible for measuring events, such as binary execution, file access, or policy changes. IMA relies on a TPM and provides integrity events that are measured and stored in the RoT, enabling hardware-based RA.

Various proposals have been made to perform integrity verification through RA of digital systems. One example is Keylime [15], which is a cloud-oriented RA framework and does not directly consider IoT scenarios. In the IoT context, some works have been proposed, such as CRAFT [16] and DRAFT [17], but they do not fully meet the requirements arising from a dynamic IoT network, as discussed in Section 2.

Contribution

This paper presents EMBRAVE, a novel TPM-based RA framework, leveraging IMA, designed to provide a dynamic and scalable solution in IoT networks. We introduce flexibility and dynamism with the design of a Join (and Leave) Protocol for new IoT devices willing to join the network. We present the novel design and corresponding implementation of EMBRAVE. We also present and describe the tests performed and the results achieved in terms of the system’s functionalities and performance.

Paper Structure

This paper is organized as follows. Section 2 provides a general overview of the background concepts and technologies, while the related work is discussed in Section 3. Section 4 discusses the EMBRAVE design, presenting the architecture and two novel protocols. Section 5 describes the threat model and provides a security analysis of the framework. Section 6 presents the open-source implementation, while Section 7 presents the experimental performance results. Finally, Section 8 presents the conclusion and future work.

## 2. Background

### 2.1. Trusted Computing

A system is considered *trusted* when it is always assumed to behave as expected. For this reason, a trusted component is also assumed to be trustworthy, and its misbehavior cannot be detected. To verify the trustworthiness of a platform’s behavior, it must be possible to measure its state to compare it with the expected correct state. The TCG defines the concept of a Trusted Platform (TP) as a system that can measure all its software components and configurations. A measure is represented by a hash value (e.g., SHA256) computed on the contents of the components. A TP requires an RoT, which is a platform element considered trusted because its misbehavior cannot be detected at runtime.

An RoT consists of three components:

1. Root of Trust for Measurement (RTM): This is responsible for measuring the platform components and storing the measurements securely. This component, also known as the Core Root of Trust for Measurement (CRTM), is often a piece of firmware stored in a one-time programmable ROM, and its purpose is to measure and store the measurement of the first mutable code that takes control of the platform.
2. Root of Trust for Storage (RTS): This is responsible for storing the measurements produced by the RTM.
3. Root of Trust for Reporting (RTR): This is responsible for the external reporting of the measurements stored in the RTS.

The TCG proposed a TP implementation that relies on an additional component called the Trusted Platform Module (TPM) [9]. The TPM is a specification defined to build a Hardware RoT (HRoT) [18]. It is implemented as a tamper-resistant chip that can store cryptographic keys and perform cryptographic operations. The new TPM 2.0 version [19] introduces crypto-agility to the hash algorithm by supporting both SHA2 and SHA3, as opposed to the previous 1.2 version which only supported SHA1.

The TPM acts as the RTS and RTR. The Platform Configuration Registers (PCRs) in the TPM implement the RTS. They store digest values, and their length is fixed to the length of the output of the associated hash algorithm. The TPM has a set of 24 PCRs, called a bank, for each supported hash algorithm. The PCRs can only be reset by a platform reset, and they can only be written using the *extend* operation. This operation stores a new digest in the PCR by hashing the concatenation of the old PCR value and the new digest:

(1) $$PCR_{new}=Hash_{alg}(PCR_{old}\;\|\;new\_digest)$$

The operation of extension is meant to keep the history of the values saved in a PCR.

The TPM also implements the RTR by providing the Quote command. This command provides the signature of the PCR values specified as input to the command. The signature is computed by the TPM using a private key stored in the TPM called the Attestation Key (AK). An AK is a signing key that is only used to sign digests generated by the TPM. The key pair can be generated at any time within the TPM, and the private part never leaves the TPM.

Another important credential managed by the TPM is the Endorsement Key (EK). The EK is a private/public key pair that is unique for each TPM and identifies the TPM; it is stored in a protected area. The certificate of the EK is stored in the TPM together with the key pair. This certificate is issued by the manufacturer and can be retrieved from the TPM.

### 2.2. TPM Software Stack (TSS)

**Figure 1:**
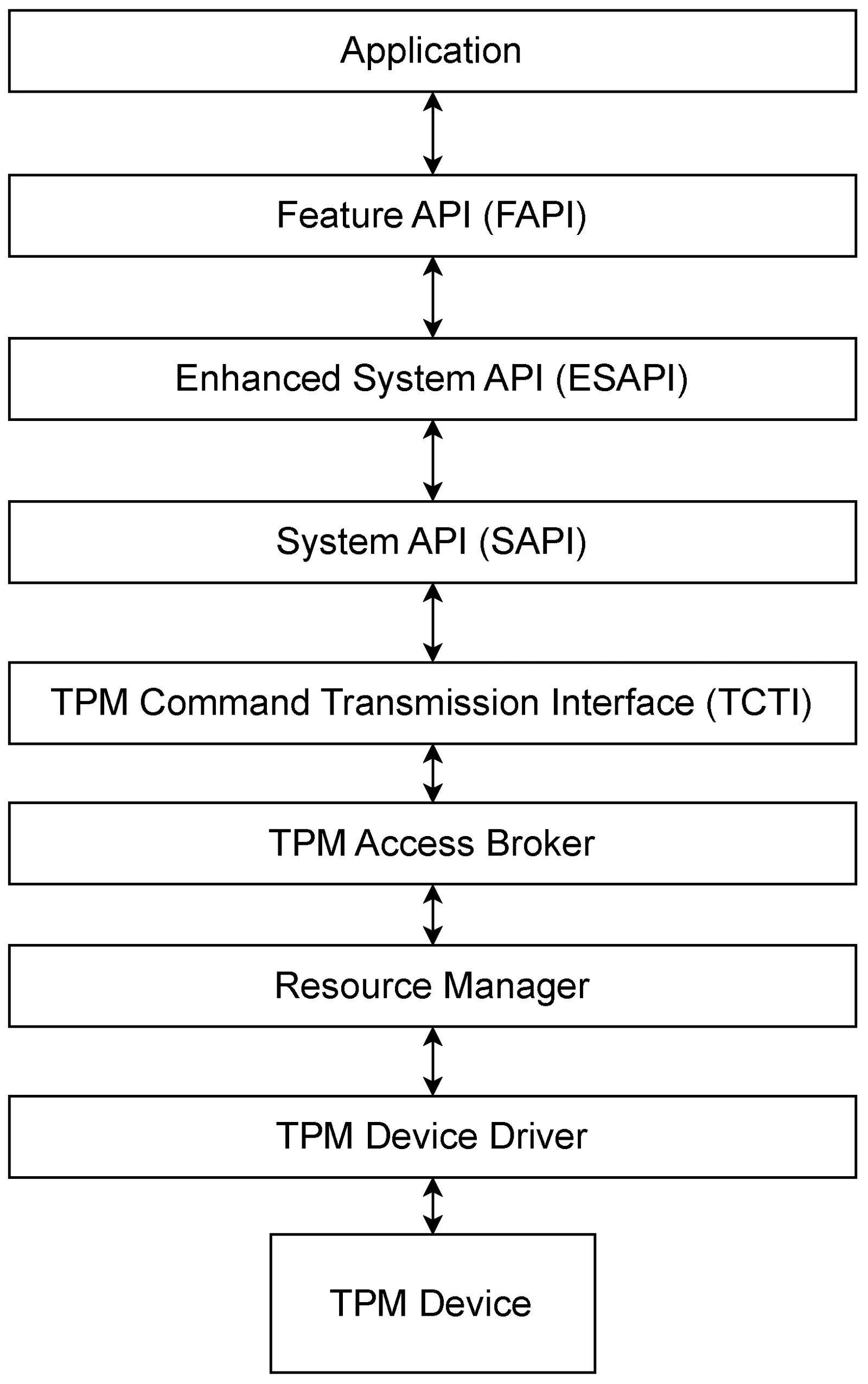
TPM Software Stack (TSS) description [20].

The TCG proposed the TPM Software Stack (TSS) [20] specification to manage the interactions of a generic application with the TPM. This specification defines the necessary software components to communicate with a TPM and exploit its functionalities. Figure 1 depicts the TSS; its specification distinguishes between the communication management components and the interfaces used by an application.

The communication management components are as follows:

1. TPM Command Transmission Interface (TCTI): This handles the communication with lower levels of the stack. This is necessary because there can be different TPM implementations (e.g., hardware TPM, firmware TPM) and each type has different interfaces.
2. TPM Access Broker: This manages concurrent accesses to the TPM, ensuring that a process accessing the TPM can complete its task without interference from other processes.
3. Resource Manager: This manages the TPM memory similar to a virtual memory manager. This is necessary due to the limited TPM resources.
4. TPM Device Driver: This is the operating system-specific driver that manages the direct communication with the TPM chip.

Three kinds of Application Programming Interfaces (APIs) offer different levels of abstraction:

1. System API (SAPI) is the lowest-layer API designed to be called from any level in a platform software stack (e.g., firmware, BIOS, OS, application);
2. Enhanced System API (ESAPI) is placed on top of the SAPI, offering cryptographically protected communication with the TPM;
3. Feature API (FAPI) is the higher-level API that hides all the low-level details and is intended to make it easier to perform operations on the TPM (e.g., key generation).

### 2.3. Secure and Measured Boot

The platform boot is a critical phase for establishing a chain of trust from the RoT to the software components running on the platform. During boot, each component takes control of the system to perform its operations and then passes it to the next one.

The secure boot [21] verifies the trustworthiness of a component before passing it control of the platform. This process starts with the RoT, which measures the next component to be executed (e.g., first-stage boot loader) and checks its trustworthiness. If the component is trustworthy, it can take control of the platform and start executing. When a boot stage is considered compromised, the boot process is interrupted and the platform freezes (i.e., if a component is assessed as untrusted, the secure boot procedure stops the boot).

The measured boot [22] differs from the secure boot in that the trustworthiness of the components is not enforced locally but is evaluated by a remote Verifier at runtime. During a measured boot, each component measures the next one and stores the measurement in the TPM. In turn, these measurements can be made available to a remote Verifier to assess whether the boot process was trusted.

### 2.4. Linux Integrity Measurement Architecture

The Linux kernel uses an integrity security module called Integrity Measurement Architecture (IMA) [13,14,23] to measure events in the system. IMA relies on the TPM as the RoT. IMA stores the event measurements, extending a specific PCR in the TPM, and generates a log file, called IMA log or IMA measurements list. This log file contains the full list of events that have occurred in the system, allowing a Verifier to recalculate the value of the associated PCR and check the software integrity of the system.

IMA is a configurable, policy-based module for measuring various types of events, such as access to memory-mapped files and binary execution. IMA uses *hooks*, which are routines attached to specific sections of kernel code, to measure events. When an event is triggered, the hook measures it, stores the measurement in the IMA log, and extends the PCR associated with IMA. The default is PCR 10, but this can be configured. Extending the PCR 10 with the new measurement provides integrity coverage for the IMA log. A remote Verifier can perform the extension operations by parsing the IMA log. If at the end of the process the value matches the value received from the TPM by the Quote command, it means that the IMA log has not been tampered with. The IMA log can then be used to analyze the events that have occurred on the platform and verify that it is behaving as expected.

Each IMA log entry is composed of several fields to identify the event that produced the measurement. The structure of the entries can be configured by selecting the *template* to use. The template [24] is a set of fields used to compose the entry. IMA provides some default templates, such as ima-ng, ima, and ima-sig. The default template is ima-ng, which is composed of the following fields:

1. PCR: The register that stores the measurement. The default is PCR 10.
2. template-hash: The hash calculated on the concatenation of the template fields that compose the entry. The PCR field is excluded from the calculation. This is the actual value extended in the PCR 10 to cover the integrity of all the information stored in the template entry.
3. template-name: The name of the template used to compose the entry.
4. filedata-hash: The hash of the file data that produced the measurement. In this case, the hash is prefixed with the name of the hash algorithm used to calculate it.
5. filepath: The absolute path of the measured file.

Table 1 shows an example of the IMA log structure with the ima-ng template. The first entry in the IMA log is always the boot_aggregate, which is calculated by applying the *extend* operation to all measurements during the boot stages (BIOS, boot loader, kernel). The result is added to the IMA log, associated with the boot_aggregate filepath, to allow a Verifier to check the measured boot process and take proper countermeasures in case of an untrusted behavior.

### 2.5. Remote Attestation

The RA [8,25] is typically implemented as a challenge–response protocol. The Verifier challenges the Attester, who in turn produces proof of its integrity for the Verifier. The Verifier compares the proof against reference values. RA techniques rely on the presence of a secure element on the device that acts as an RoT. The main specifications for building RoTs are the TPM and DICE [11].

#### 2.5.1. Single-Device RA

In this scenario, the Verifier directly contacts each platform in the IoT network for which integrity verification is required. The proposed approaches can be classified into three main categories: hardware-based, software-based, and hybrid.

##### Hardware-Based Attestation

This family of techniques relies on some specific hardware extensions or a dedicated secure element on the Attester (e.g., the most widely adopted secure element for RA is the TPM). Other techniques are based on CPU hardware extensions to implement the concept of the Trusted Execution Environment (TEE) [26], such as Intel SGX [27] or the latest introduced Intel TDX [28] and ARM TrustZone [29].

##### Software-Based Attestation

Hardware-based solutions are the most secure. However, this approach may not always be practical due to hardware and software constraints, especially in resource-constrained IoT platforms. For these reasons, software-based solutions have been proposed [30]. Pioneer [31] is an example of a software-based primitive that does not rely on CPU architecture extension or any secure co-processor. SWATT [32] is another software-based solution where the Verifier is required to have precise information about the Attester, like the clock speed and the Instruction Set Architecture (ISA). The solution presented in [33] is designed to provide a more precise attestation, covering the device’s memory to protect against return-oriented programming attacks.

##### Hybrid Attestation

In certain networked environments, software-based RA methods may not provide adequate security due to potential threats from adversaries [34]. To mitigate these risks, there are hybrid solutions that use both software and hardware, such as SMART [35], which requires minimal hardware modifications to embedded MCUs. HAtt [36] exploits Physical Unclonable Functions (PUFs) to protect secrets on IoT platforms and to attest different regions of the platform’s memory.

#### 2.5.2. Collective RA

Collective RA techniques may be used when a group of Attesters wants to prove their integrity to a Verifier. The main challenge is to reduce the communication overhead between the Verifier and the Attesters. These techniques can be classified based on the basic architecture and target of the attestation process—in this case, there are tree-based [37,38] and distributed aggregation techniques [39,40]—or by considering the network topology—in this case, there are solutions for dynamic [41,42,43] or static [44,45] network topology. These techniques allow the integrity of a group of platforms (swarm) to be verified by contacting only one platform, which in turn collects the integrity proof of the group and forwards it to the Verifier. A disadvantage of these techniques is that it is typically not possible to know the integrity status of a specific platform but only of a group (swarm) of devices.

## 3. Related Work

Keylime [15] is an RA framework, based on the TPM 2.0 or its virtual implementation (vTPM) [46], for software integrity verification in an Infrastructure as a Service (IaaS) cloud environment. Keylime addresses the typical challenges of an IaaS trusted computing system like multiple owners of the system and the presence of virtualization technologies. Even though Keylime is a complete and solid framework, it has a different goal than EMBRAVE, which focuses on dynamic IoT networks in contraposition with Keylime, which is oriented to cloud environments.

CRAFT [16] (Continuous Remote Attestation Framework for IoT) is designed to work in dynamic networks with moving IoT platforms. CRAFT does not define an RA protocol but leverages existing protocols. The framework covers the offline and online phases. In the offline phase, CRAFT defines the initial parameters, including the security parameters. In the online phase, CRAFT defines the flow of messages for integrity verification through protocol-defined packets. CRAFT targets IoT scenarios by adding a level of abstraction but relies on other RA frameworks to work properly. Despite the high flexibility added by CRAFT by supporting several technologies and RA techniques, it reduces the security of the process because it is not based on a solid hardware RoT and can accept devices not supporting strong security features. EMBRAVE instead is a complete framework composed of IoT platform management protocols and the RA protocol.

DR@FT [17] (Dynamic Remote Attestation Framework and Tactic) is an RA framework with a flow-based attestation. It follows the principles defined by the TCG and leverages the capability of the TPM in combination with IMA. It uses TPM version 1.2 and IMA on the Linux kernel version 2.6.26-rc8, an old and outdated kernel version compared to the new LTS versions (v5.15, v6.1). This system, although it proposes a complete implementation, has become obsolete due to the technologies it uses. It supports the TPM 1.2 as a hardware RoT, which provides strong security properties, but it is an outdated version of the specification, which people are discouraged from adopting. In contrast, EMBRAVE leverages the capabilities of the TPM 2.0 and relies on the latest features of the IMA Linux submodule.

The authors of [47] proposed an IoT Device-trusted Remote Attestation Framework. It is an RA framework for real-time monitoring of massive attacks on IoT platforms to prevent remote control and zombification. It supports various IoT platforms equipped with TPM and different TEEs. In this case, the framework does not support a dynamic environment. In addition, the presence of a hardware RoT is not guaranteed, and this can significantly reduce the security of the RA process for those devices. To address this aspect, EMBRAVE defines specific protocols that provide the ability to manage high dynamics in the composition of the IoT network.

Another solution proposed in the literature is HYDRA (Hybrid Design for Remote Attestation) [34], which, differently from EMBRAVE, is a hybrid RA design, which leverages mostly software features to provide security. In particular, it exploits the microkernel seL4 [48], which, being formally verified, guarantees correctness in the software implementation. Despite the formal verification, seL4 is a software component, and for this reason, the security level provided is lower than a hardware solution. In this particular case, for example, the exclusive access to the AK of the device is guaranteed by the microkernel, with no additional hardware protection. On the other hand, EMBRAVE leverages the secure TPM device to protect the AK and guarantee its secrecy and binding with the device.

HAtt (hybrid remote attestation) [36] is a hybrid RA solution for IoT devices. It aims to reduce supplementary resources to perform integrity verification, such as a TPM, in order to reduce costs. It leverages a Physical Unclonable Function (PUF) in order to protect the secrets. A PUF is a hardware module which permits deriving secure values without having to store them in any storage, reducing the risk of leakage. Unlike a TPM, an AK derived from a PUF still needs to be memorized in memory when in use, which exposes it, if not protected in some other way, to some potential attacks.

The WISE [39] framework, differently from EMBRAVE, is oriented to collective (swarm) attestation. In this case, the framework aims to perform attestation of large IoT device networks by reducing the overhead of single-device attestation. The main constraint of this solution is that the network is required to be static, because it must be configured previously, knowing the network topology, limiting the fields of application. Differently, EMBRAVE, with the defined Join Protocol, supports dynamic changes in the topology of the network, managing devices that join and leave the network.

In Table 2, we report a comparison based on the framework capabilities in terms of security, suitability for IoT and embedded systems, and management of dynamic network topology changes.

## 4. EMBRAVE Design

### 4.1. General Architecture

EMBRAVE is a TPM-based RA framework oriented to dynamic IoT networks where IoT platforms can join and leave the network at any time.

**Figure 2:**
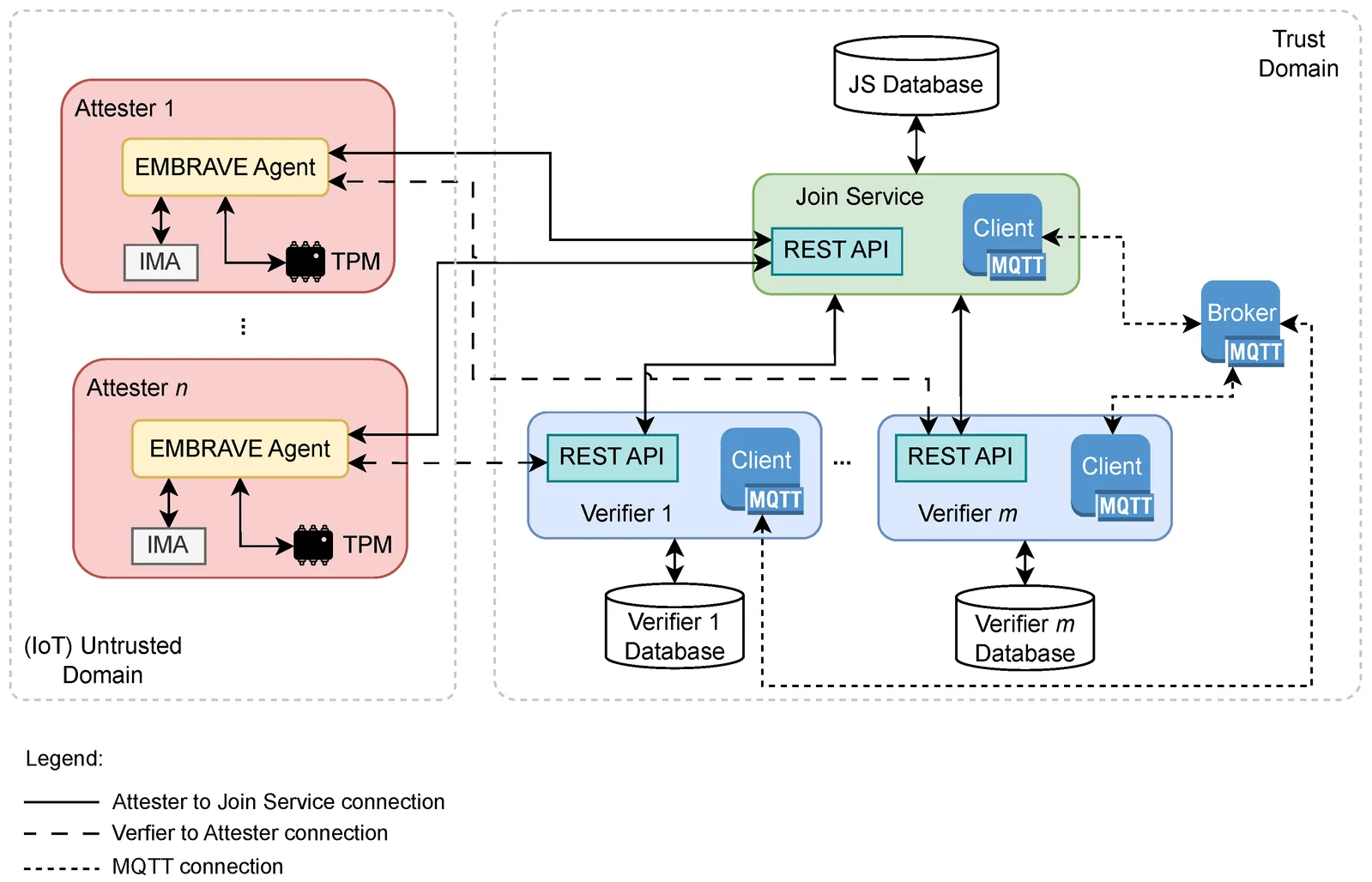
EMBRAVE modular architecture.

Figure 2 shows the modular architecture of EMBRAVE, where multiple Attesters can be attested and multiple Verifiers can be deployed. The architecture comprises an *Untrusted Domain*, where all IoT platforms reside, and a *Trust Domain* under the admin control, where the Join Service and Verifiers reside. The communication between components is managed with REST APIs and MQTT messages. REST APIs are mainly used to communicate outside and inside the Trust Domain, while MQTT is used only in the Trust Domain.

The Join Service, the EMBRAVE Agents installed on the IoT platforms, the *Verifiers*, and the MQTT Broker are the main components of the EMBRAVE architecture. These components interact in compliance with two protocols: a novel Join Protocol and the Remote Attestation Protocol compliant with TCG recommendations [49].

The following sections describe the role of each component and present newly designed interactions between them.

#### 4.1.1. Join Service

The Join Service is the main component of the EMBRAVE architecture. It is responsible for managing Join and RA procedures, following the protocols presented in Section 4.2 and Section 4.3. It is the entry point for new IoT platforms (Attesters) to join the network. Each new Attester must present itself to the Join Service to join the framework. Once the Join Service has verified the authenticity of the platform, it selects one of the active Verifiers to challenge its trustworthiness. This component is also responsible for managing the registration phase of a Verifier. Each newly deployed Verifier must contact the Join Service, which adds it to the pool of active Verifiers. The Join Service stores all the data relating to the registered Attesters and Verifiers in a database. In particular, it stores data about the status of new Attesters during the Join Protocol, described in Section 4.2. It also ensures that information about the deployed Verifier is retained in case the Join Service is restarted.

The Join Service exposes a set of REST APIs to manage the Attester and Verifier registration:

1. POST /api/request_join allows a new Attester to join the network. When receiving a request on this API, the Join Service generates a challenge and sends it back to the Attester.
2. POST /api/confirm_credential allows a new Attester, after having called
  /api/request_join, to resolve the challenge received and to send the result back to the Join Service.
3. POST /api/request_join_verifier allows a newly deployed Verifier to join the network in the Trust Domain.

The Join Service maintains the credentials and integrity status to build a global view of the IoT network’s trustworthiness.

#### 4.1.2. EMBRAVE Agent

The EMBRAVE Agent is a software component deployed on the Attester. It directly interacts with the TPM to generate the necessary credentials to join the network and to run the TPM2_Quote command on the TPM and answer attestation requests.

When the EMBRAVE Agent starts, it first creates a resident AK for signing the attestation quotes. Then it retrieves the EK certificate stored in the TPM. Note that more than one EK certificate can be available in the TPM, each one associated with a different algorithm. The certificate read by default is the one associated with an Elliptic Curve Cryptography (ECC) algorithm. If no ECC certificate is found, the EMBRAVE Agent reads the certificate associated with the RSA algorithm, which is always present. The EMBRAVE Agent uses this data to contact the Join Service, which in turn uses it to assert the authenticity of the Attester’s TPM. After this phase, the EMBRAVE Agent remains running, waiting for the attestation requests from the assigned Verifier.

The EMBRAVE Agent exposes the /api/quote API, which is used by a Verifier to ask for an attestation report composed of the result of the TPM2_Quote command and the IMA log during the RA protocol.

#### 4.1.3. Verifier

The Verifier is the component responsible for challenging and evaluating the trustworthiness of an Attester. The Verifier interacts with the EMBRAVE Agent on the Attester.

A Verifier’s first action is to join the framework by notifying the Join Service, as it is important for the Join Service to maintain the list of active Verifiers to allocate the RA tasks. The Verifier then waits for the Join Service to assign it a new Attester, which must be periodically attested. Each Verifier maintains a dedicated database to store the information about the Attesters assigned to them. This database stores all the information the Verifier needs to verify the integrity of a specific Attester, such as its keys and the whitelist location reference. The database is also used to maintain the state of the Verifier in the event of a restart so that the Verifier can continue to attest the assigned Attesters.

The communication between the Join Service and a Verifier is mainly managed with MQTT messages, as described in Section 4.1.4. A Verifier also exposes the /api/still_alive API, which is used by the Join Service to rebuild the list of already active and registered Verifiers after a restart.

#### 4.1.4. MQTT Broker

We use MQTT [50] for communication and notification within the Trust Domain. We defined two parametric topics:

1. attest/<verifier-id>: The Join Service publishes on this topic to notify a Verifier that a new Attester successfully joined the network and must be attested. A Verifier subscribes to the topic constructed with its identifier. Upon the successful joining of a new Attester, the Join Service publishes on the MQTT topic verfier-id of the Verifier selected for starting the periodic attestation of the Attester.
2. status/<verifier-id>: All registered and active Verifiers publish on this topic to share the integrity report with the Join Service after each remote attestation of an Attester. The integrity report contains all the information related to the integrity status of an Attester with its trustworthiness evaluation.

#### 4.1.5. EMBRAVE Scalability

The main objective of the framework is to provide a lightweight environment, mostly on the Attester side. Nevertheless, the possibility of having a large number of IoT platforms in the network could cause an overload on the Verifier side. The Verifier has no special requirements, giving the administrator the freedom to deploy it on the server infrastructure of their choice based on the expected attestation load. This freedom allows the administrator to scale vertically, increasing the Verifier’s computing resources. In addition, to support the case where vertical scaling is not desired, EMBRAVE supports the simultaneous use of multiple Verifiers for horizontal scaling. Verifiers can be dynamically added and removed from the framework to cope with the attestation load. Scalability is introduced by design, allowing the network/infrastructure owner to increase or decrease the number of Verifiers according to the expected or actual load. Increasing the number of Verifiers allows for a more distributed attestation load, as new Attesters are always assigned to the Verifier with the least number of active attestation processes. In particular, a new Attester is only assigned to one Verifier to keep the number of attestation processes as evenly distributed as possible across all active Verifiers.

### 4.2. Join Protocol

We designed a Join Protocol to support dynamic networks, where (guest) IoT platforms can frequently join and leave the network. The protocol involves four steps for an Attester to join the network. First, the Attester sends its EK certificate to the Join Service, which in turn verifies the authenticity of the Attester’s TPM. Second, the Join Service sends a challenge to the Attester in response to the successful EK certificate verification. The challenge is consumed by the Attester to prove the possession of the previously sent credentials. Third, the Attester sends the resolved challenge back to the Join Service. Finally, the Join Service selects a Verifier from the pool of active Verifiers and triggers the start of the periodic RA procedure.

**Figure 3:**
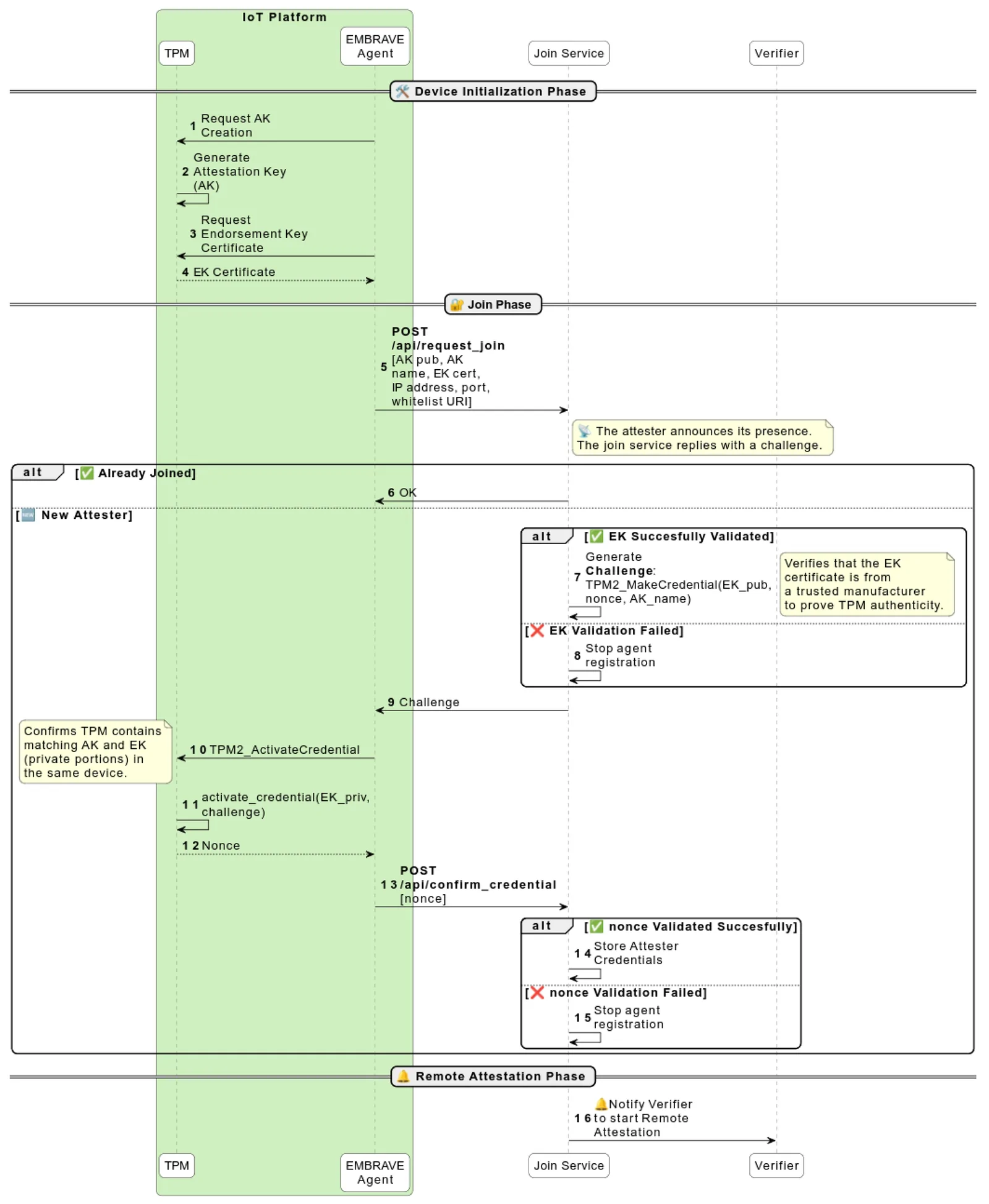
Join Protocol.

Figure 3 details the interactions of the Join Protocol. When the EMBRAVE Agent starts, it checks that the platform has an IP address assigned for a configured network interface. If the check is successful, it starts the EMBRAVE Join Protocol as described hereafter.

The EMBRAVE Agent creates the AK (1), (2) and retrieves the EK certificate (3), (4) from the non-volatile memory of the TPM. The first choice is the ECC certificate; if not present, the RSA certificate is retrieved. If some of these steps fail, the Agent will not be able to provide to the Join Service all the necessary data needed for a correct authentication of the device, and for this reason, the registration phase will fail. In this scenario, the Agent is not considered trusted because no attestation has been performed and the authentication of the device failed during the Join phase. In this case, the administrator of the network can choose the remediation mechanism that best suits the considered application scenario and act accordingly. Once all credentials have been successfully retrieved, the EMBRAVE Agent contacts the Join Service (5), requesting to join the network using the /api/request_join API.

The EMBRAVE Agent sends the following data to the Join Service:

1. The IP address and port to which the Verifier must send requests during the RA protocol;
2. The EK certificate;
3. The AK public part;
4. The AK name provided as the output of the AK generation command. It is calculated by hashing the AK public part with the hash algorithm associated with it:
  (2) $$AK_{name}=Hash_{alg}\left(AK_{publicPart}\right)$$
  The derivation of the name is defined in the TPM 2.0 structure specification [51].
5. The whitelist URI. The whitelist is the list of trusted files and their measures on the IoT platform. They are measured in a protected environment. It is provided as a URI representing a local or remote location. For example, it may be the name of the database stored in the Verifier (pre-shared) or a secure URL where the Verifier can retrieve the list (e.g., certified by the manufacturer).

When the Join Service receives the data from the Attester, it first verifies the validity of the EK certificate (EK_cert) (6) to assert the authenticity of the Attester TPM.

It then executes the TPM2_MakeCredential command (7) to prepare a challenge for the Attester to prove the following: (i) that it owns the AK private part, (ii) that it owns the EK private part, and (iii) that the AK key pair resides in the same TPM associated with the EK certificate. The TPM2_MakeCredential takes as input the EK public part, the AK name, and a secret that the Attester must find to prove its knowledge. The secret in our design is a random *nonce*. The challenge is sent back to the Attester (8), and this puts the Attester in a pending state until it sends back an answer.

After receiving the challenge, the Attester runs the TPM2_ActivateCredential command (9), (10) on the TPM with the challenge as input. The command will extract the correct nonce (11) from the challenge in response if the credentials used to produce the challenge are the correct ones.

The Attester sends the answer back to the Join Service (12) via the

/api/confirm_credential API. The Join Service compares the Attester’s answer with the secret nonce and accepts the join request if it matches or rejects it otherwise.

In the case of success, the Join Service stores the Attester credentials (13) and notifies a Verifier (14) to start the RA protocol on that specific Attester by sending to the Verifier its credentials.

### 4.3. Remote Attestation Protocol

**Figure 4:**
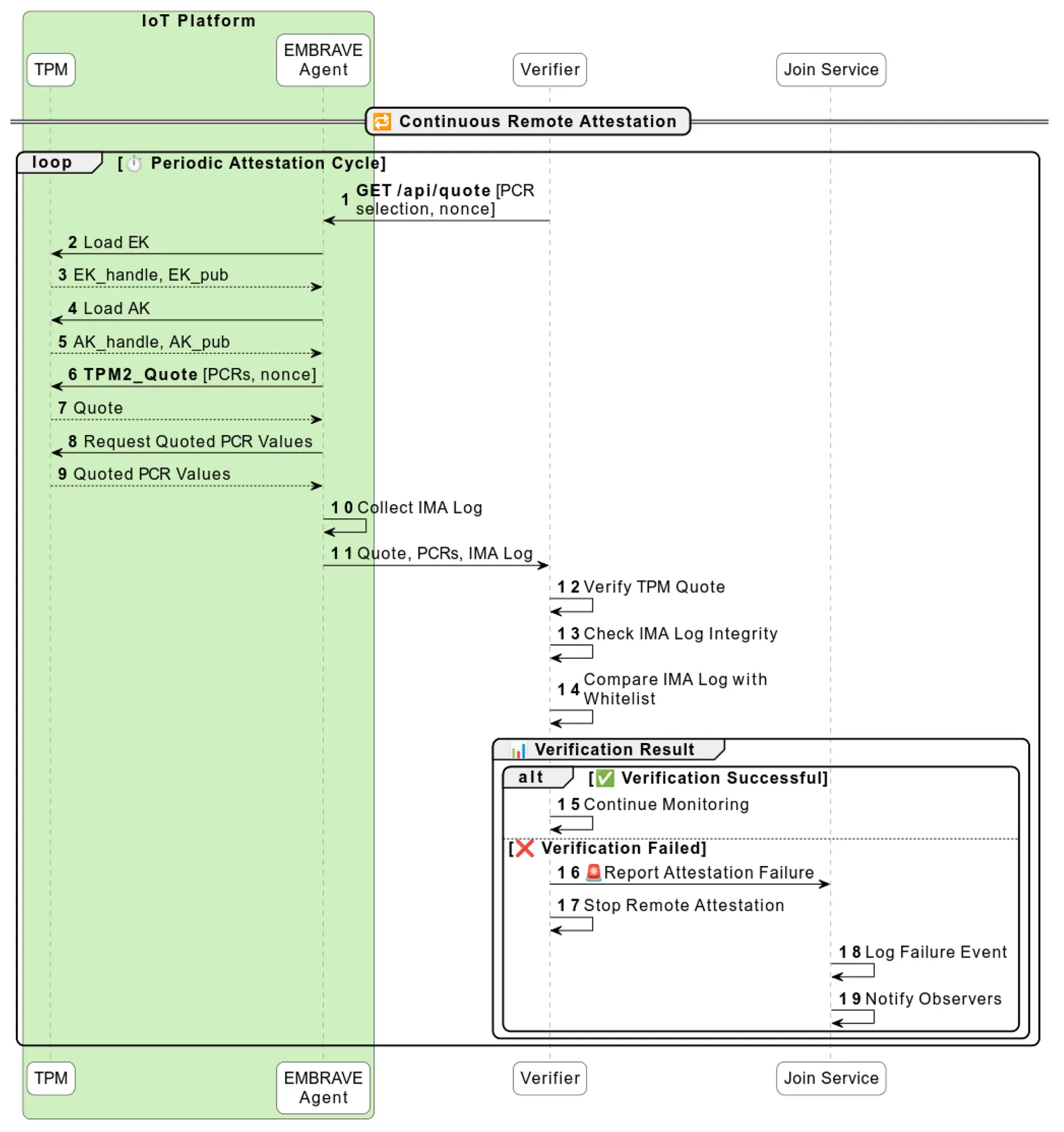
Remote Attestation Protocol.

The RA protocol shown in Figure 4 starts immediately after the Join Protocol has been successfully completed. At the end of the Join Protocol, the Join Service selects a Verifier and notifies it to start the periodic attestation of the new Attester.

The Join Service publishes a message on the topic verifier/<verfier-id>, which the selected Verifier listens to. In turn, the Verifier adds the Attester’s information to its database and begins periodic attestation of the Attester to verify its trustworthiness.

The Verifier contacts and challenges the Attester (1) through the /api/quote API of the EMBRAVE Agent.

The Verifier generates a random *nonce* and sends it to the EMBRAVE Agent. This *nonce* is used by the TPM2_Quote command of the TPM to generate a fresh quote, which protects from reply attacks. When the EMBRAVE Agent receives the RA request, it loads into the TPM memory (2), (3), (4), (5) the necessary credentials (i.e., EK and AK) and performs the following operations:

1. Generates a quote with the TPM2_Quote command (6), (7). The output of this command is the signature of the values of the selected PCRs with the AK private part.
2. Reads the quoted PCR (8), (9) values with the TPM2_PCR_Read command.
3. Reads the IMA log (10) containing all the runtime measurements.
4. Sends the quote, the values of the quoted PCRs, and the IMA log back to the Verifier (11).

In turn, the Verifier checks the quote (12) against the AK public part received from the Join Service and the PCR values received from the Attester. If this control succeeds, the verification of the integrity of the IMA log starts (13). This verification requires the Verifier to simulate the PCR 10 extension for each entry in the log file as follows:

(3) $$template\_hash=Hash_{Alg}(field_{1}\;\|\;\ldots \;\|\;field_{n})$$

(4) $$PCR_{new}=Hash_{Alg}(PCR_{old}\;\|\;template\_hash)$$

If the simulated PCR 10 coincides with the PCR 10 value received from the Attester, the Verifier can start comparing each measure against the whitelist associated with the Attester to analyze the software integrity. The failure of one of these verifications implies marking the Attester as untrusted.

We also designed an incremental delivery strategy for the IMA log for efficiency. The Attester sends to the Verifier only those entries that have not been verified in the previous RA round. This strategy reduces the number of bytes sent to the Verifier and speeds up the overall attestation process. The Verifier checks the integrity of the IMA log during the first RA round and securely stores the value of the simulated PCR 10 and the size of the IMA log already verified. In the next RA round, the Verifier requests only the IMA log entries from the Attester, starting with the entry immediately after the last one checked. To verify the incremental IMA log, the Verifier simply restarts the PCR 10 extension simulation from the previously stored value.

### 4.4. Leave Protocol

An IoT platform may dynamically leave the network without notifying the Join Service or the Verifier. The attestation performed by the Verifier is a periodic process, and if an IoT platform has left the network, it will be unreachable for attestation requests from the Verifier. After a configurable number of failures, the Agent is declared untrusted. When the attestation result is ready, it is published on the specific MQTT topic. This report can contain all the information that can be useful for remediation after an integrity verification failure. In this case, the cause of having marked the Attester as untrusted is the unreachability, in particular, after the configured number of retries by the Verifier. The threshold for retries before marking a device as untrusted can also be variable, based on the period of attestation. Depending on this period, the threshold can be set to 1 if the attestation period is on the order of tens of seconds, also based on the average network and device load. On the other hand, it can be placed at a higher value if the attestation period is on the order of a few seconds or eventually if the average device load is high, making some connection failures acceptable. To have a more accurate threshold for retries before marking an Attester as untrusted, as future work, an adaptive mechanism can be added, which can consider several aspects, such as network metrics and specific use case policy.

## 5. Security Analysis

### 5.1. System Scope

The main objective of our design is to enable continuous integrity verification of IoT platforms in a dynamic network. Moreover, our design enables guest IoT platforms (i.e., platforms that are not under the full control and ownership of the network admin) to join the network as well.

The design assumes that each IoT platform integrates a correct and valid implementation of a TPM 2.0 compliant with the TCG specification [19] and that the TPM manufacturer correctly generated and certified the EK of the TPM. Note that the TPM can also be implemented as a firmware version, as proposed in [52,53], without compromising the use of the EMBRAVE framework.

In addition, the design assumes that each IoT platform in the network is equipped with a valid CRTM, such as a shielded boot ROM, which starts the process of secure boot [21] and/or measured boot [22] to measure the first-stage firmware. The secure boot is meant to enforce the correctness and trustworthiness of the running components on the IoT platform, while the measured boot enables remote verification of the boot sequence trustworthiness. The secure boot process is required for all guest IoT platforms to enforce the correct boot of the platforms, hence the integrity of their software stack up to the kernel. The integrity of the applications is then verified via an RA protocol, using the integrity measurements provided by IMA.

### 5.2. Threat Model

We consider adversaries that aim to tamper with IoT platforms by remotely manipulating their software: installing and executing malicious code or changing platform configurations and policies. More specifically, we place attackers on the Attester side, outside the Trust Domain. Thus, a Verifier is always considered a legitimate entity in our architecture.

Finally, we do not consider physical attacks that aim to manipulate IoT platforms by exploiting physical channels such as the MPU, external buses, and the TPM chip.

### 5.3. Security Considerations

EMBRAVE aims to protect an IoT platform from the first boot stage to the application layer. Secure boot enforces the correct boot sequence by verifying the signature of a component from the very first firmware stage. Enforcement is achieved because the signature verification fails if a boot component is compromised, stopping the process and halting the platform. This process covers the entire software stack, up to the kernel. Once the kernel boots correctly, IMA is initialized before the first userspace process starts and the first kernel module is loaded. Thus, IMA starts measuring from the very first integrity event.

The security properties that EMBRAVE guarantees are as follows:

1. AK Unforgeability: No adversary can produce a valid signature using $AK_{privatePart}$ without access to the associated physical TPM.
2. Freshness and replay protection: The TPM quote is guaranteed to produce always fresh values and not reusable ones thanks to a *nonce*.
3. Measurement soundness: PCR values correctly reflect the current platform integrity state at the time of attestation request, also thanks to the freshness of the quote.

**Figure 5:**
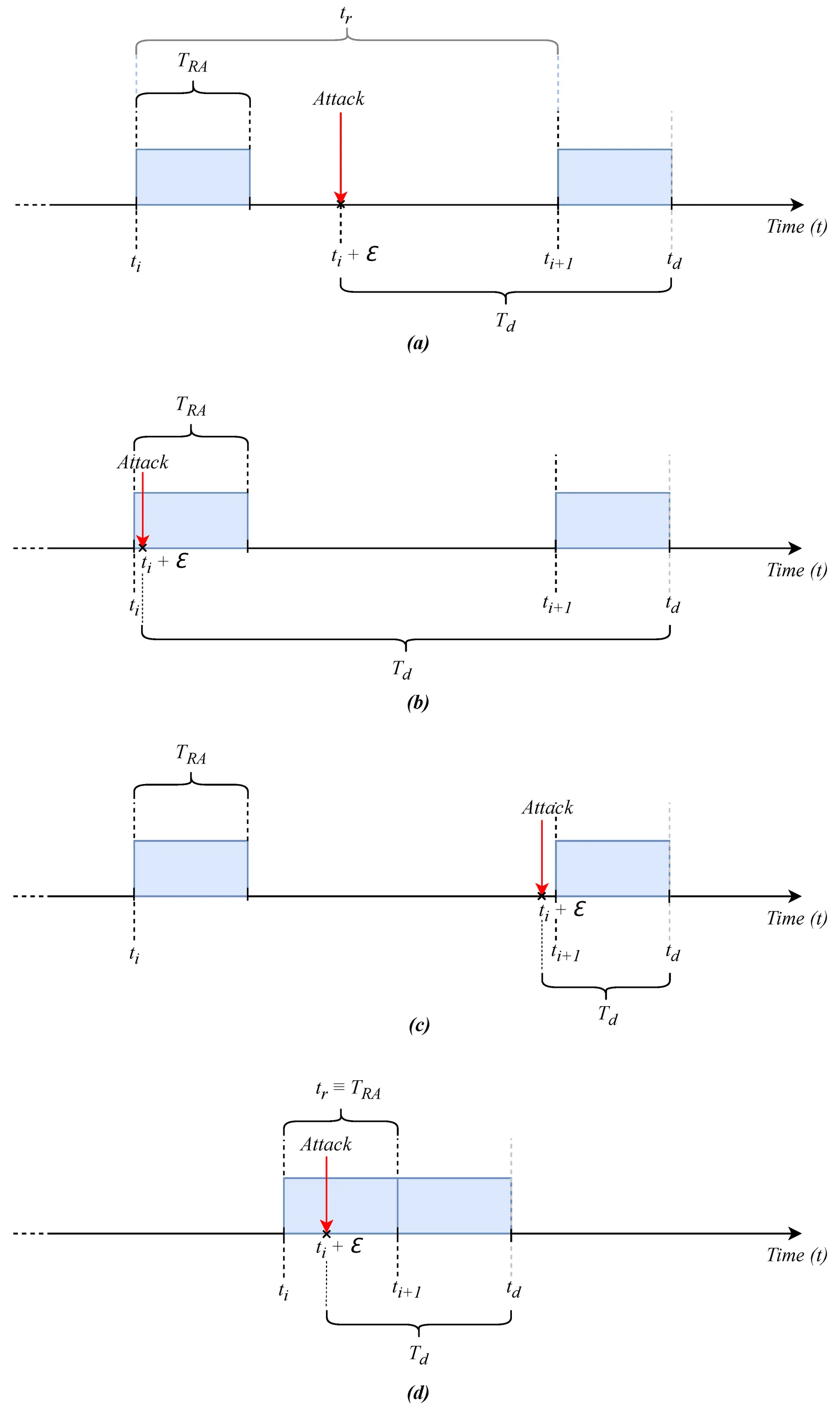
(**a**) Generic attack scenario. (**b**) Worst-case scenario for attack detection time. (**c**) Best-case scenario for attack detection time. (**d**) The best configuration for minimizing the attack detection time.

Figure 5 shows different attack scenarios. Figure 5a represents a generic scenario where the attack is performed between an attestation round, starting at $t_{i}$, and the subsequent round, starting at $t_{i+1}$. The attack happens at time $t_{i}+\epsilon$ with the conditions $\epsilon >0,\;t_{i}+\epsilon <t_{i+1}$. Defining $t_{r}$ as $t_{r}=t_{i+1}-t_{i}$, the configured time between two attestation rounds, and $T_{RA}$ as the time for performing a complete attestation, the time $T_{d}$ to detect the attack is estimated by $T_{d}=\left(t_{r}-\epsilon \right)+T_{RA}$.

Considering the worst scenario, depicted in Figure 5b, where the attack happens right after the beginning of an attestation round (with ϵ>0 small as desired), the time to detect the attack is the largest and is estimated by

(5) $$T_{d}=\left(t_{r}-\epsilon \right)+T_{RA}$$

Considering the best scenario, depicted in Figure 5c, where the attack happens right before the beginning of an attestation round, the time to detect the attack is the smallest and is estimated by

(6) $$T_{d}=T_{RA}+\epsilon$$

Note that the above equations represent the upper and lower limits for the detection time:

(7) $$T_{RA}<T_{d}<t_{r}+T_{RA}$$

The latter equation suggests that we must properly configure $t_{r}=T_{RA}$ to minimize the upper limit, as depicted in Figure 5d. In this case, the time to detect the attack is

(8) $$T_{d}<2T_{RA}$$

**Figure 6:**
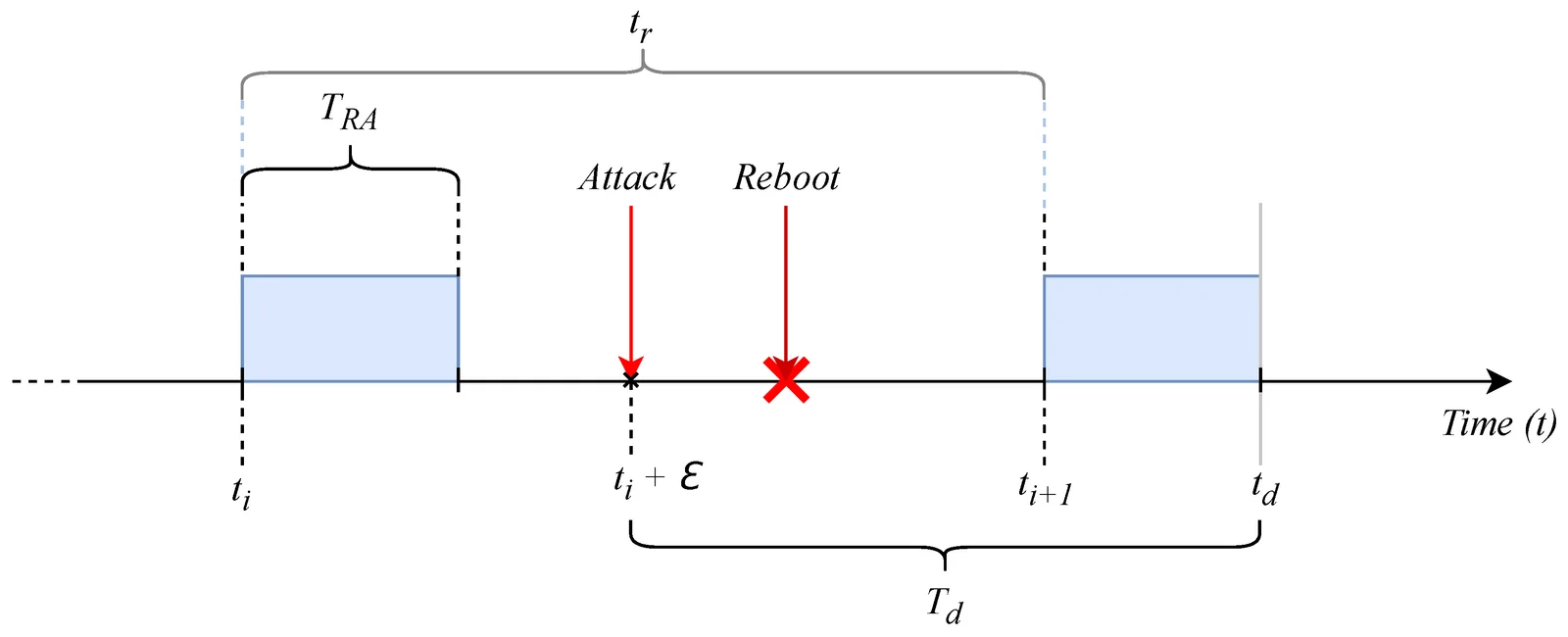
Attack scenario where the attacker tries to hide the compromise by rebooting the device, causing the reset of the IMA log.

We also consider the possible attack scenario where the attacker performs a platform reboot right after having compromised the device (Figure 6). The reboot is performed to reset the IMA log and the status of the PCR 10 in the TPM. This malicious attempt is still detected thanks to the implementation of the incremental IMA log mechanism. With the incremental IMA log mechanism, the Verifier would ask the Attester only the new measurements in the IMA log, and the measurements would not pass the simulated extension of the PCR 10.

Another important consideration is about the new Join Protocol. During the Join phase, initiated by an Attester, the IP address is associated with an AK. This makes it possible to verify the following: (i) that all Attesters in the network can be attested correctly, and (ii) that there are no misconfigurations (e.g., the same AK associated with more than one IP address). However, during the Join phase, a malicious platform can act as a man in the middle (misbinding attack) and intercept the Join request of another platform. As a result, the malicious platform would be able to associate its IP address with an uncompromised platform. The malicious platform can then forward an attestation request from the Verifier to the uncompromised platform and send a valid integrity report to the Verifier. In this scenario, there would be an issue in the list of devices connected to the network, as the IP address of the uncompromised device would not be associated with an AK and could be detected, indicating a possible problem in the overall integrity of the network.

In our threat model, the Verifier is considered a trusted entity, but in analyzing different scenarios, we present some comments on possible security countermeasures for protecting sensitive data. In particular, it would be crucial to guarantee the integrity and authentication of the used whitelists. Representing the reference values used to evaluate the trustworthiness state of an Attester, a whitelist represents critical data if the threat model changes and the Verifier is not considered trusted. In this case, it is necessary to protect them from possible malicious compromises. This can be achieved by providing digitally signed whitelists. To manage the trusted public keys for verifying the whitelists, Linux Keyrings [54] can be used that permit storing and using keys in kernel space without exposing them to userspace and malicious actors. The keys can be loaded at the reset time of the device, adding the possibility to seal the keys in the TPM. In this way, they would be loaded only if the device boots in the expected state. In this case, any manipulation of the device software image would prevent the keys from being loaded. Also, introducing encryption of the Verifier database can increase the level of security when changing the threat model. Some Format-Preserving Encryption techniques [55] can be used in order to reduce the overhead in accessing the data during the RA process and minimizing the overhead. In this way, the data related to the Attesters would also be protected against possible attackers who aim to manipulate them in order to mark as trusted a compromised device. This can also be considered for the Join Service database, in a scenario where it is not considered trusted. Some specific access control and authentication mechanisms can be adopted in order to allow only authorized entities to manage sensitive data. To this end, label-based Mandatory Access Control (MAC) mechanisms can be used, such as SELinux [56], that allow only a subset of processes, correctly marked with labels, to access specific resources. This permits the system to evaluate access permission on every process. In this way, even if the process has root privilege, it cannot access a resource if it does not have the correct label.

### 5.4. Attack Simulation

To evaluate the effectiveness of the attestation process, we here simulate a scenario where an attacker loads a malicious driver in the device, as depicted in Figure 5a.

Given the ima_policy=critical_data [57], the IMA log is as shown in Algorithm 1, reporting the measurement (shown in italics) of the correct driver loaded during the boot phase.

When the attacker loads the malicious driver, this causes a new measurement in the IMA log because it is different from the previous one, and the cache mechanism of IMA leads to a miss. After loading the malicious driver, the IMA log correctly reports the new measurement (in bold) as shown in Algorithm 2.

**Algorithm 1** IMA Log example showing expected kernel module measurement (in italic).
[…] sha256:d51f[…]60098 boot_aggregate[…] sha256:1711[…]03987 kernel_version 362e313[…]63312b[…] sha256:13e4[…]aaa23 init_module adee572[…]123e43[…] sha256:aee4[…]31312 init_module cc09eef[…]550aff[…] sha256:a400[…]009e3 init_module 093dd37[…]d36440…*[…]* *sha256:1711[…]a0098* *init_module* *10eedf0[…]009ade*…


**Algorithm 2** IMA Log example showing expected kernel module measurement (in italic) and the compromised kernel module (in bold).
[…] sha256:d51f[…]60098 boot_aggregate[…] sha256:1711[…]03987 kernel_version 362e313[…]63312b[…] sha256:13e4[…]aaa23 init_module adee572[…]123e43[…] sha256:aee4[…]31312 init_module cc09eef[…]550aff[…] sha256:12ae[…]ddda3 init_module 109aacc[…]a680ee[…] sha256:a400[…]009e3 init_module 093dd37[…]d36440…*[…]* *sha256:1711[…]a0098* *init_module* *10eedf0[…]009ade*…
**[…] sha256:ee45[…]14009 init_module a550bec[…]110eee**
…


The Verifier detects the attack because even if the simulated extension of the PRC 10 coincides with the real PCR in the TPM, the check of the IMA log against the golden values fails.

## 6. Implementation

We implemented EMBRAVE in the C programming language, and the open-source code can be found at [58].

The implementation uses the tpm2-tss library [59], an open-source implementation of the TCG TSS [20] specification, to interact with the TPM chip. Specifically, we used the ESAPI to implement the interactions between the EMBRAVE Agent and the TPM chip, achieving the best compromise between complexity and flexibility. The use of the EASPI permitted the integration of complex operations, such as AK creation, in the code base of the system without relying on other components. In this way, it has been possible to obtain a small code footprint, minimizing the impact on the device memory.

To retrieve the IMA log, the virtual filesystem interface provided by the module is used. In particular, the IMA module offers, in its specific directory and with the structure placed in the securityfs virtual filesystem [60], two interfaces (highlighted in bold in Algorithm 3) for reading the measurements list. These two interfaces permit reading of the measurements list in ASCII format and in binary format. We used the binary format for reading the IMA log and for sending it to the Verifier. This permits the reduction of data in transmission by almost 30% of the dimension of the IMA log compared with the ASCII representation. Using the binary format also permits the removal of the overhead of converting file measurements from hexadecimal format, reducing the verification time on the Verifier side and reducing the minimal RA period $T_{RA}$.

All cryptographic operations leverage the open-source OpenSSL library [61]. We also used the Mongoose [62] open-source library to implement the REST API and MQTT client functionalities. This library is maintained for the long term and is explicitly designed and developed for embedded devices. We did not implement a new MQTT Broker. We adopted the Mosquitto [63] implementation for testing purposes. EMBRAVE does not impose any MQTT Broker implementation requirements.
**Algorithm 3** Listing of the IMA directory in the securityfs virtual filesystem.$ ls -la /sys/kernel/security/integrity/imatotal 0drwxr-xr-x 2 root root 0 […] .drwxr-xr-x 4 root root 0 […] ..-r- -r- - - - – 1 root root 0 […] **ascii_runtime_measurements**-r- -r- - - - – 1 root root 0 […] **binary_runtime_measurements**- -w- – – - - 1 root root 0 […] policy-r- -r- - - - – 1 root root 0 […] runtime_measurements_count-r- -r- - - - – 1 root root 0 […] violations

## 7. Experimental Evaluation

### 7.1. Testbed Description

The testbed comprises two IoT platforms and a laptop to deploy all components in the Trust Domain of the EMBRAVE architecture. The two IoT platforms are as follows:

1. A VAR-DT8MCustomBoard V2.1 [64] (Variscite, Lod, Israel) equipped with a DART-MX8M-PLUS V1.2 [65] running at 1.8 GHz and 4 GB RAM, using FSLC Wayland with XWayland 4.0 (kirkstone) [66], with Linux kernel version 5.15.60.
2. A Raspberry Pi 4 Model B [67] (Raspberry Pi, Cambridge, UK) with the processor running at 1.8 GHz and 4 GB RAM, using Raspbian GNU/Linux 11 (bullseye) with Linux kernel version 5.15.79.

**Figure 7:**
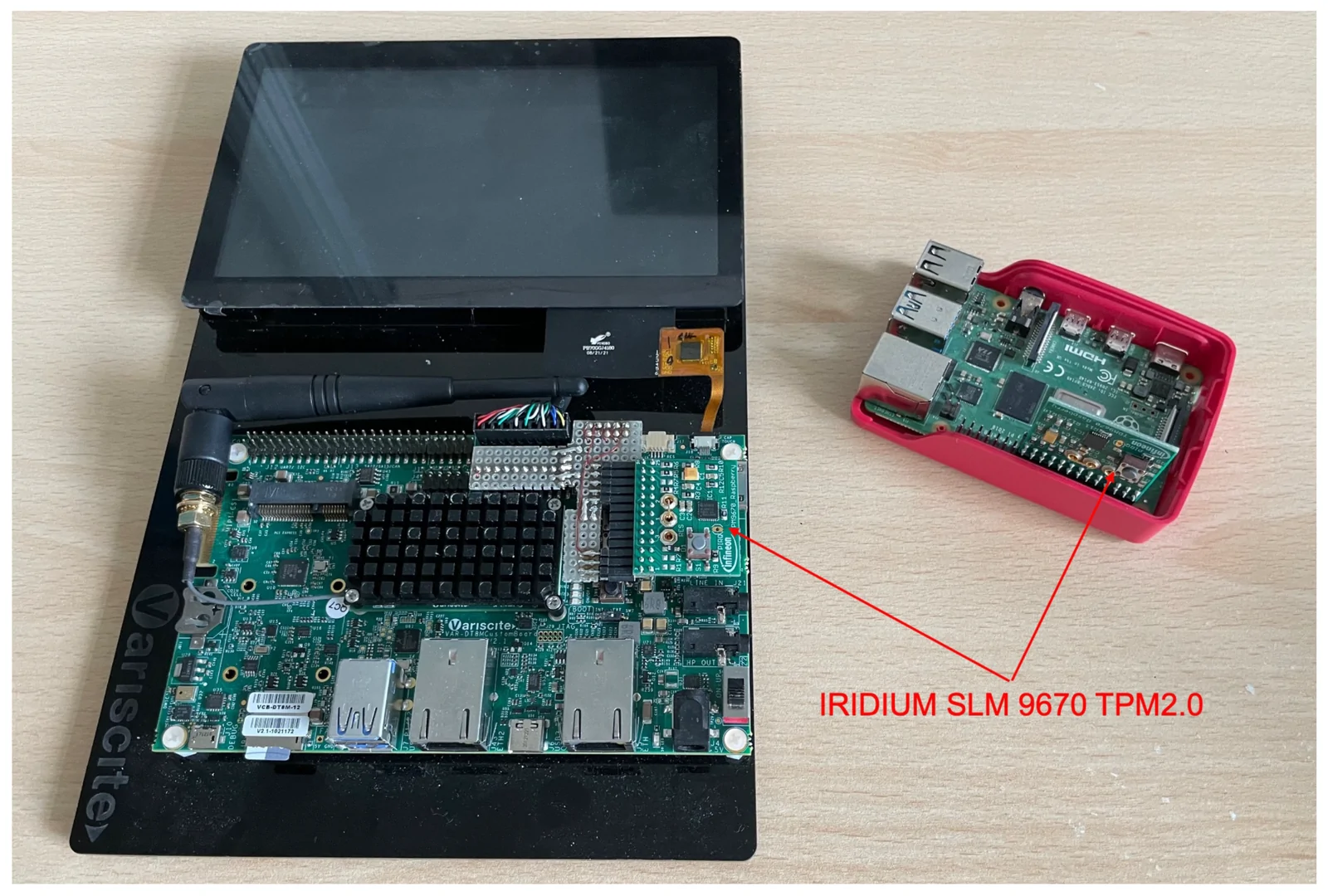
VAR-DT8MCustomBoard V2.1 mounting an IRIDIUM SLM 9670 TPM2.0 (**left**), Raspberry Pi 4 Model B mounting an IRIDIUM SLM 9670 TPM2.0 (**right**).

Both platforms are equipped with an external board providing a TPM 2.0 as shown in Figure 7. The TPM board is an IRIDIUM SLM 9670 TPM2.0 [68] (Infineon, Munich, Germany), mounting an OPTIGA™ TPM SLM 9670 [69] (Infineon, Munich, Germany) chip. We also set the tcb IMA policy for both devices.

We deployed the Verifier, the Join Service, and the MQTT Broker on a DELL Latitude 5440 laptop equipped with an Intel^®^ Core™ i7-1355U 1.70 GHz CPU and 16 GB of RAM. These services ran in a virtual machine with Ubuntu 22.04.1 (6.5.0 Linux kernel) installed.

In a real-world scenario, the IMA log tends to reach a stable state where the measurement log almost stops growing because all the most frequently accessed files have already been measured. In addition, the contents of files do not change very often, so even if they are accessed again, they will not trigger a new IMA measurement.

To evaluate EMBRAVE in high-attestation-load scenarios where the IMA log grows, we used a script that ran in an infinite loop. In each cycle, the process slept for a random amount of time, between 2 and 8 seconds, and then produced a new measurement in the IMA log. This choice also allows us to compare the performance of the full IMA log and incremental IMA log strategies.

To first evaluate the performance of EMBRAVE, we measured the time required to complete the Join Procedure and the remote attestation, evaluating also the incremental IMA log feature. In this case, we performed the test with two physical devices equipped with a TPM device in order to simulate real-scenario devices that join the framework and the remote attestation performed on real devices. For each test, we performed the measure 1000 times and calculated the mean of these to obtain the average time:

(9) $$\mu =\frac{1}{N}\sum_{i=1}^{N}x_{i}$$

where $x_{i}$ represents the single measure and *N* the number of time measures. In addition to verifying the time measures, we also evaluated the standard deviation:

(10) $$\sigma =\sqrt{\frac{1}{N}\sum_{i=1}^{N}{\left(x_{i}-\mu \right)}^{2}}$$

We also performed a scalability test on the Join Service in order to evaluate its performance in the case of several concurrent Join Procedures being requested. For this test, we exploited a virtual testbed in order to simulate an increasing number of concurrent Agents that request to join the framework. In this case, we evaluated several latency metrics to obtain a more comprehensive view of the performance. We evaluated the average latency (avg) alongside median (p50), 90^th^ percentile (p90), and 99^th^ percentile (p99) latencies. Percentile metrics characterize the distribution of response times, reflecting different aspects of system responsiveness.

Specifically, the p50 denotes the maximum latency experienced by the fastest 50% of Join Procedures, thus representing the typical or the “average” time for a device to join the framework. Another analyzed metric is the p90, which indicates the latency threshold below which 90% of Join Procedures complete, revealing the performance experienced by the majority but capturing moderate slowdowns. Finally, the p99 represents the latency threshold below which 99% of Join Procedures complete, thus highlighting very few but significantly delayed responses that can increase the time needed to obtain the trustworthiness state of a new device. Formally, the latency at percentile $p_{x}$ is defined as follows:

(11) $$Latency_{p_{x}}=\max\left\{L|P\left(L_{join}\leq L\right)\leq \frac{x}{100}\right\}$$

where $Latency_{p_{x}}$ is the latency at percentile *x*, *L* represents latency values, and $P(L_{join}\leq L)$ denotes the proportion (probability) of a device joining whose latency $L_{join}$ is less than or equal to *L*. In addition, we also evaluated the error rate of the Join Service to verify if, at some concurrency level, the Join Service can miss some Agent registration requests and then fail in adding them to the framework.

### 7.2. Experimental Results

**Figure 8:**
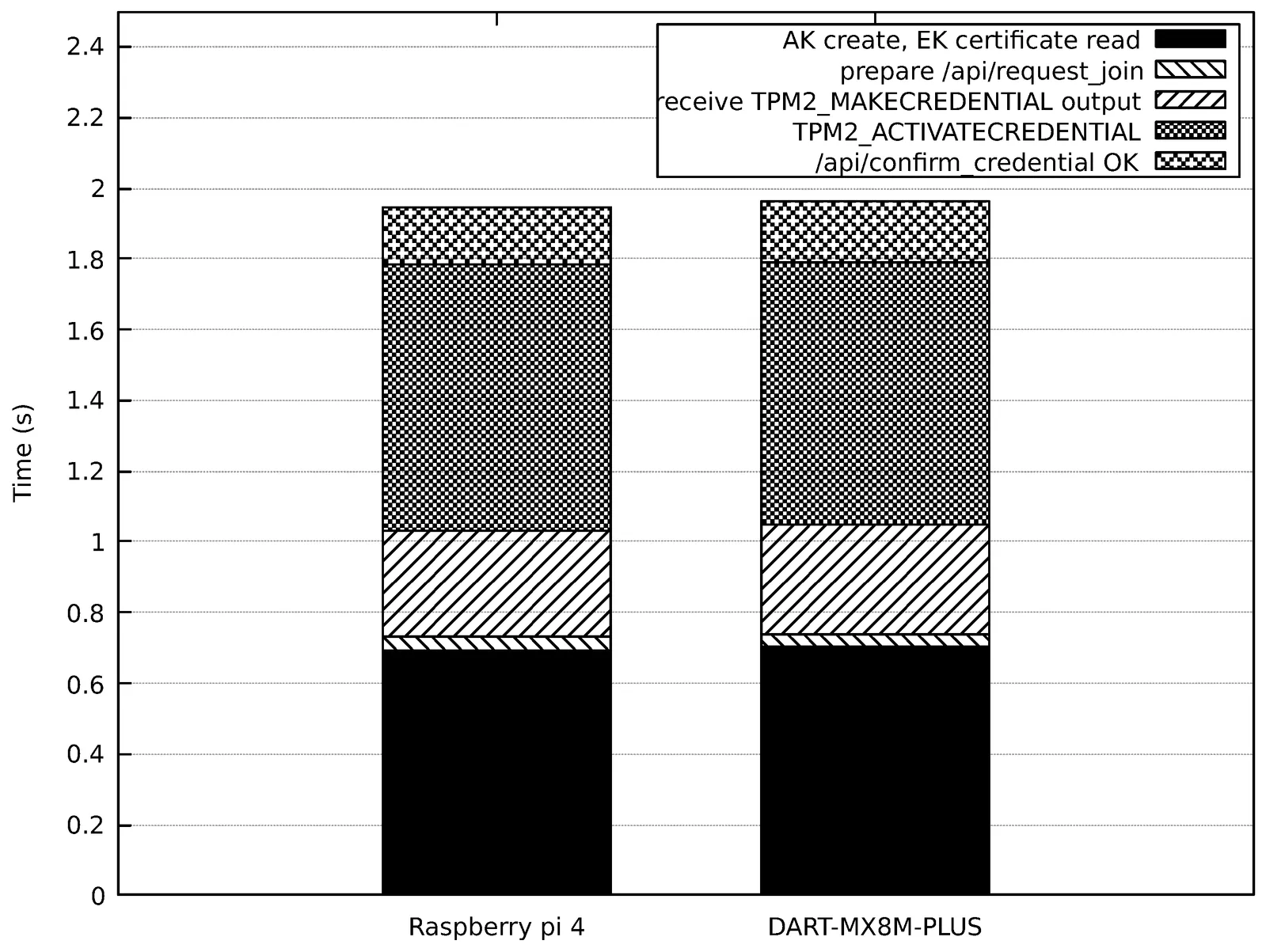
Time to complete the Join Procedure.

Firstly, we measure the time each platform requires to join the network. Figure 8 shows the time spent in each phase of the Join Protocol. It is possible to appreciate that 74% of the overall time is spent performing TPM operations both on the Raspberry Pi and on the VAR-DT8MCustomBoard. This is caused by the limited resources of the TPM. For this reason, the time spent on the entire process is very similar between the two platforms. The time needed by the Raspberry Pi is 1.95 s and by the VAR-DT8MCustomBoard is 1.97 s. For the time measures performed, the standard deviation for the Raspberry Pi was 0.12 and for the VAR-DT8MCustomBoard was 0.14, indicating consistent values.

**Figure 9:**
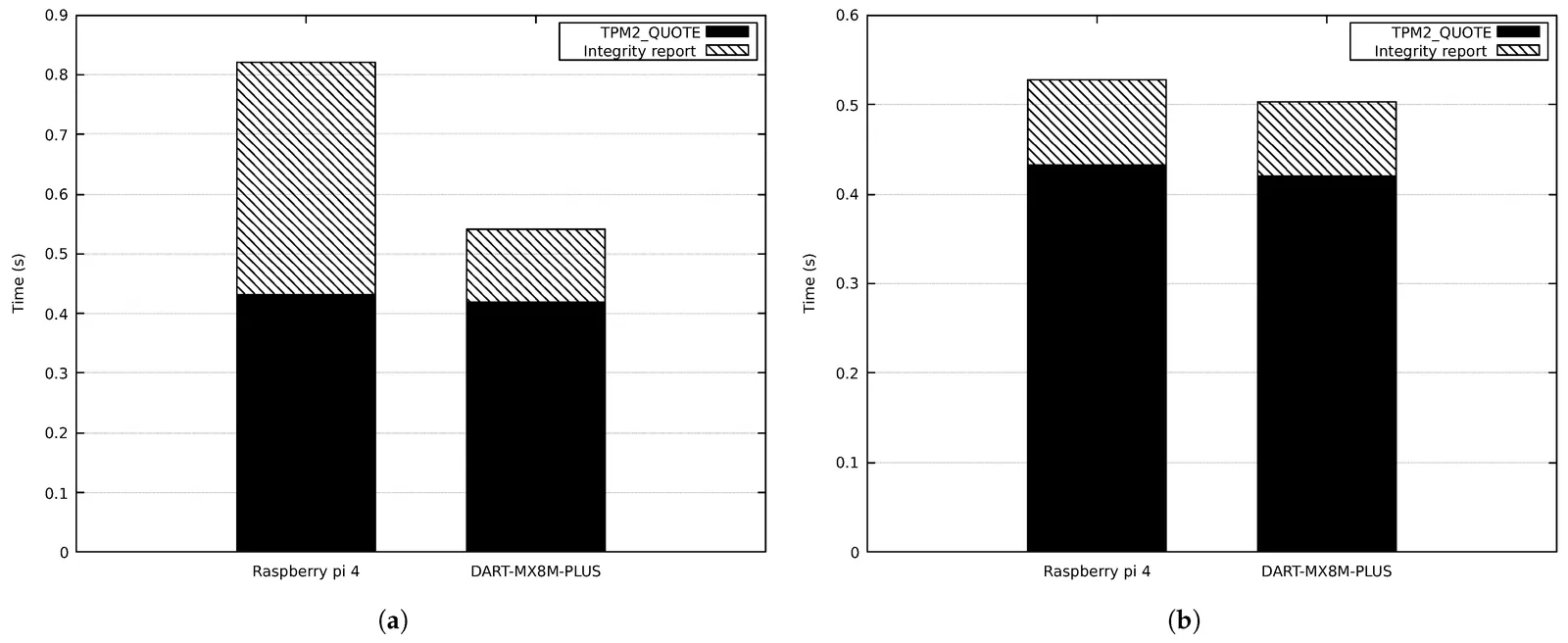
Time for an RA round evaluating the entire IMA log (**a**). Time for an RA round evaluating the incremental IMA log (**b**).

Secondly, we measured the time for an RA round $T_{RA}$ to evaluate the performance of the RA protocol and to provide quantitative results useful to complement the considerations addressed in Section 5.3. We first evaluated the case when the entire IMA log is sent at every RA round. Figure 9a shows the time required to evaluate the entire IMA log. In both platforms, the time spent to perform a TPM2_Quote is on average 0.4 s. In terms of total time, the difference between the two platforms is due to the configuration of the operating systems installed. The one installed on the VAR-DT8MCustomBoard is lighter than the one installed on the Raspberry Pi. This implies fewer measurements performed by IMA and so a shorter IMA log to send to the Verifier. The IMA log on the VAR-DT8MCustomBoard is about 100 kB, and the Raspberry Pi one is about 130 kB. The overall time needed to perform an RA round on the Raspberry Pi is 0.82 s, while on the VAR-DT8MCustomBoard, it is 0.54 s. We defined the overhead of an attestation framework as the time to perform an attestation round minus the time spent by the TPM to prepare the quote. The time spent on calculating the quote is not framework-dependent but TPM-dependent.

We also evaluated the impact of adopting an incremental IMA log strategy. Figure 9b shows the time required by the RA protocol when only the new entries of the IMA log are sent at each round.

In the case of the VAR-DT8MCustomBoard, the time required is about 0.50 s, so there is an improvement but with a small impact. This is due to the shorter IMA log generated by the system configuration. In the case of the Raspberry Pi, the improvement is more significant because the time required is 0.53 s.

Comparing the performance of EMBRAVE with another solution based on TPM RoT demonstrates the advantages of EMBRAVE when applied to IoT networks. We reproduced the RA round test with the Keylime framework [15] to have a direct comparison with EMBRAVE. We measured the time between an RA request from the Verifier and the moment the Verifier decides on the trustworthiness state of the Attester. The test reported an average attestation time of about 0.99 s, higher than the 0.50 s achieved with the incremental IMA log strategy and the 0.82 s achieved with the entire IMA log strategy in EMBRAVE.

For an analysis on the scalability of the framework, we performed other tests in a virtual environment. This permitted the evaluation of the scalability and reliability of the framework, in particular, the Join Protocol defined. To perform these tests, a physical machine was used with the following characteristics:

1. CPU: Intel^®^ Core™ i9-13900H 5.4 GHz;
2. RAM: 32 GB;
3. OS: Ubuntu 22.04.5 LTS (Linux v6.8.0).

**Figure 10:**
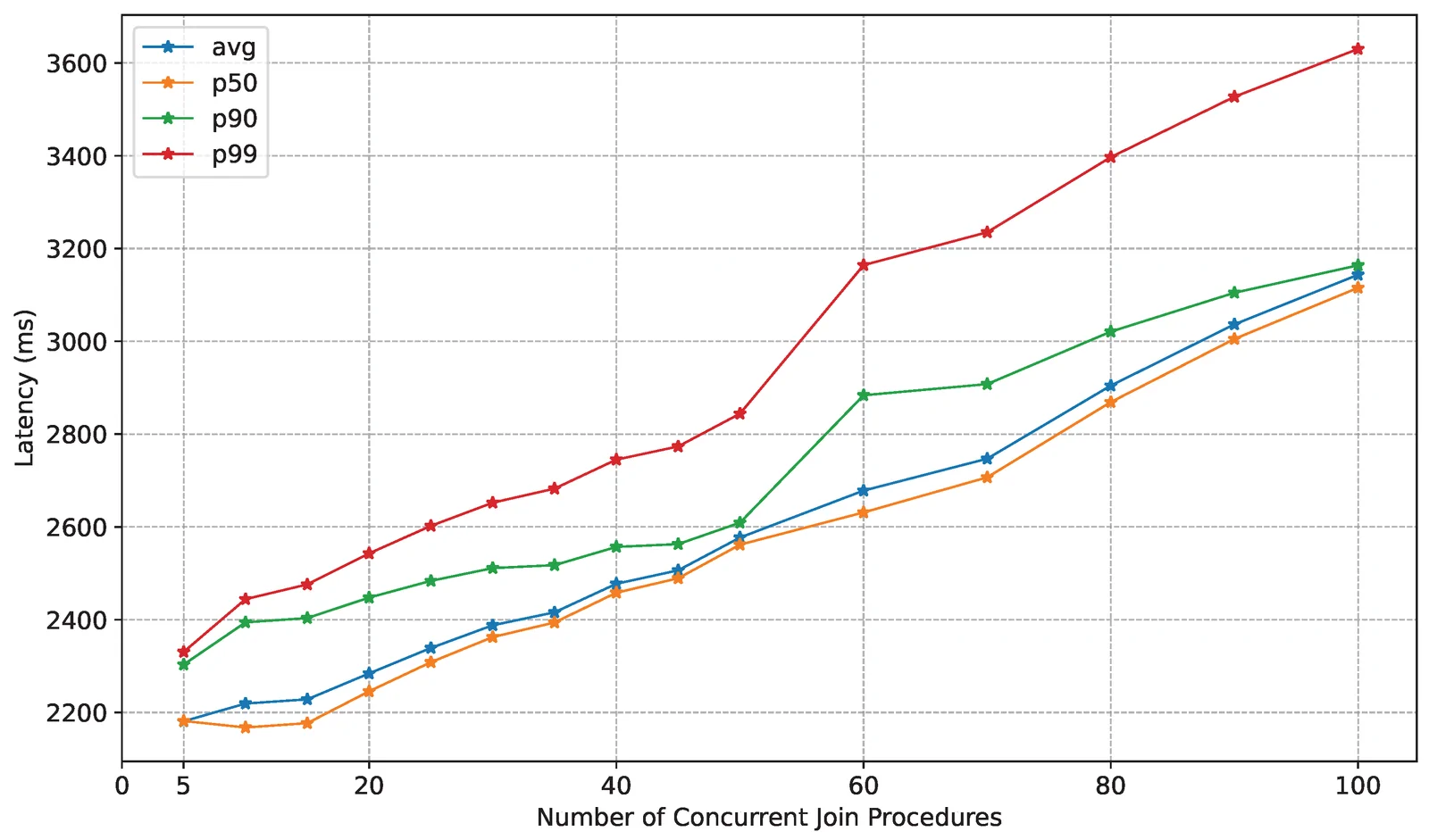
Analysis of the latency considering an increasing number of concurrency requests to the Join Service, showing (avg), median (p50), 90th percentile (p90), and 99th percentile (p99) latencies.

The Join Service was then deployed in a virtual machine with four vCPUs and 16 GB of RAM. The initial test aimed to evaluate the performance of the Join Service considering an increasing number of Agents that request to join the framework. Figure 10 provides a detailed graphical representation of the latency behavior considering an increasing concurrency level, depicting average latency, p50, p90, and p99 latencies. The graphic depicted shows that the latency of the Join Procedure linearly increases with the concurrency of requests. In particular, it is possible to notice that p50 and p90 follow the behavior of the average latency, demonstrating high scalability of the service, even in very stressed conditions.

**Figure 11:**
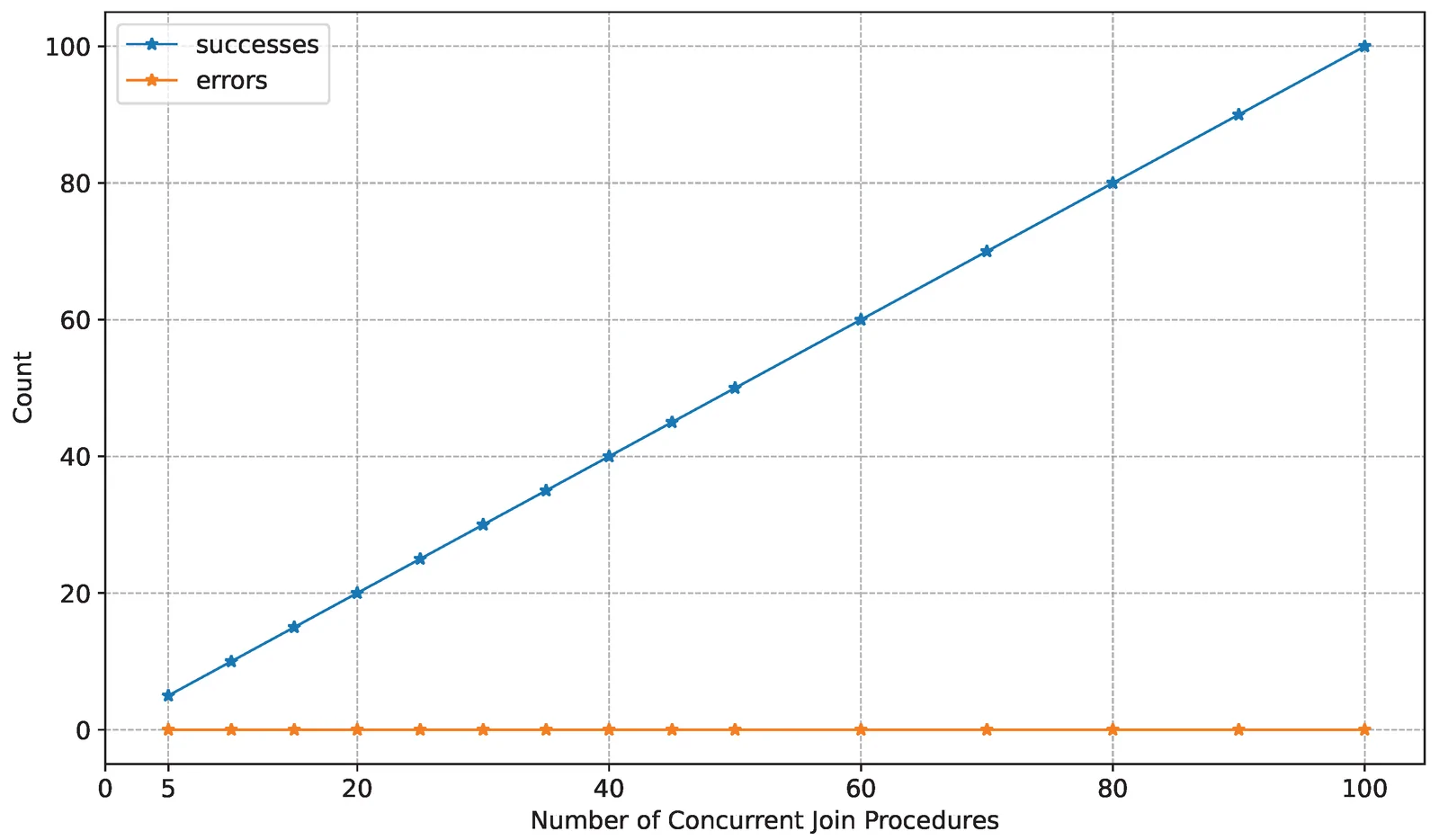
Number of succeeded and failed Join Procedures, considering an increasing number of concurrent Agents that request to join the framework.

We also evaluated the reliability of the Join Service, measuring the error rate during the concurrency test. Figure 11 shows that the error rate stays at its optimal value with up to 100 devices joining the framework concurrently. This result demonstrates a very high reliability of the service, guaranteeing its ability to serve all the requesting devices at a high level of network load.

## 8. Conclusions and Future Work

Increasing threats to IoT networks mean not only digital damage but also physical damage and human injury. Therefore, software integrity monitoring becomes critical to verify that IoT platforms behave as expected. Trusted computing techniques, in particular remote attestation, can address this critical need.

This paper has presented EMBRAVE: EMBedded Remote Attestation and Verification framEwork. EMBRAVE is a TPM-based RA framework with a modular architecture to serve dynamic IoT networks. EMBRAVE has the following features: (*i*) lightweight Join and Leave Protocols to support a high level of dynamism while binding the Join Protocol to the credentials of the TPM integrated with the IoT platforms, and (*ii*) an efficient RA protocol.

The experimental tests demonstrated its effectiveness, usability, and remarkable performance in terms of scalability and reliability, especially for the new Join Protocol defined. The metrics evaluated showed that the Join Service scales efficiently with the number of concurrent Join Procedures initiated. The obtained error rate also shows a high level of reliability of the service, demonstrating its effectiveness in very stressful scenarios.

EMBRAVE is open source and ready for extensions and improvements. Future work will focus on improving the framework, with priorities including the development of appropriate metrics and functionality for the Join Service to assign new Attesters to Verifiers based on their load and capabilities. Also, an adaptive mechanism can be introduced to determine the threshold of maximum retries during the Leave Protocol before defining an Attester as untrusted. Future work may also be directed towards enabling the authentication of Verifiers with the Join Service, adding the ability to extend the Verifier pool with external services. In addition, future work will focus on extending the support for the MARS specification from TCG and DICE implementations, such as the Open Profile for DICE proposed by Google [70].

## Figures and Tables

**Table 1:** IMA log structure with ima-ng template.

PCR	Template-Hash	Template-Name	Filedata-Hash	Filepath
10	b9[..]6a	ima-ng	sha256:99[..]17	boot_aggregate
10	10[..]e0	ima-ng	sha256:91[..]34	/init
10	60[..]cd	ima-ng	sha256:12[..]48	/usr/bin/sh
10	51[..]b6	ima-ng	sha256:d8[..]43	/usr/../libc.so
…	…	…	…	…

**Table 2:** Comparative analysis of state-of-the-art RA frameworks regarding provided key capabilities.

Framework	Framework Capabilities		
	Hardware RoT-Based	IoT- and Embedded Systems-Oriented	Dynamic Join and Leave for Devices
** Keylime [15]**	Yes (TPM 2.0)	No (cloud-oriented)	No (manual RA initialization)
** CRAFT [16]**	Not guaranteed (heterogeneous devices)	Yes	Flexible (heterogeneous RA protocols)
** DR@FT [17]**	Yes (TPM 1.2)	No (generic-purpose- systems-oriented)	No (manual registration)
** Kim et al. [47]**	Not guaranteed (heterogeneous devices)	Yes	No (manual registration)
** HYDRA [34]**	No (seL4 micro- kernel)	Yes	No (manual registration)
** HAtt [36]**	Partial (PUF-based)	Yes	No (manual registration)
** WISE [39]**	Not guaranteed (heterogeneous devices)	Yes	No (static network topology)
**EMBRAVE**	Yes (TPM 2.0)	Yes	Yes (Join Protocol defined)

## Data Availability

The original contributions presented in this study are included in the article. Further inquiries can be directed to the corresponding author.
